# Higher colistin dose during continuous renal replacement therapy: look before leaping!

**DOI:** 10.1186/s13054-015-0951-4

**Published:** 2015-06-08

**Authors:** Patrick M. Honore, Rita Jacobs, Inne Hendrickx, Elisabeth De Waele, Viola Van Gorp, Herbert D. Spapen

**Affiliations:** ICU Department, Universitair Ziekenhuis Brussel, Vrije Universiteit Brussel University, 101, Laarbeeklaan, 1090 Jette (Brussels), Belgium

We read with great interest the comments of Rocco and colleagues on colistin dosing in a recent letter in *Critical Care* [1]. The application of continuous renal replacement therapy (CRRT) indeed offers an opportunity to prescribe very high colistin doses without exposing the patient to excess toxicity [2]. However, caution is needed before implementing this so-called CRRT ‘shield’ function.

First, only hyperadsorptive filters, such as the novel acrylonitryle 69 surface treated (AN69 ST (Baxter, Lyon, France)) membrane, do serve this purpose. With this type of membrane, colistin clearance increases by adsorption at both its (rapidly saturated) surface and (less easily saturated) bulk [3]. Early saturation is unlikely, and frequent membrane changes (for example, every 12 to 24 hours) are not necessary [3, 4]. This is corroborated by our experience in a small cohort of patients who tolerated colistin doses of as high as 4.5 million IU three times a day for more than 5 consecutive days. Colistin toxicity was not observed and membrane changes were never needed during the treatment period [2, 4, 5].

Second, the functional capacity of the dialysis membrane must remain guaranteed. For this purpose, citrate is the anticoagulant of choice. Regional citrate anticoagulation has been shown to effectively counteract clogging, which ensures optimal long-term porosity [3] and, in the case of the AN69 ST filter, preserves adsorptive capacity of the membrane.

In conclusion, safe and adequate use of a higher colistin dosing regimen during CRRT requires a hyperadsorptive dialysis membrane combining convection with surface/bulk adsorption under citrate anticoagulation to consolidate membrane function.

## Abbreviations

AN69 ST: Acrylonitryle 69 surface treated
CRRT: Continuous renal replacement therapy
